# Motivational interviewing to increase drug checking and reduce overdose rates among people who use drugs: protocol for a hybrid type 1 effectiveness–implementation trial of an adjunctive intervention

**DOI:** 10.1186/s12889-025-24460-y

**Published:** 2025-09-30

**Authors:** Katie Bailey, Steffanie A. Strathdee, Angela R. Bazzi, Tara Stamos-Buesig, Morgan Godvin, Alicia Harvey-Vera, Daniela Abramovitz, Carlos F. Vera, Thomas L. Patterson, Peter J. Davidson, Annick Bórquez

**Affiliations:** 1 School of Medicine, University of California San Diego, 9500 Gilman Dr., La Jolla, San Diego, CA 92093 USA; 2 Herbert Wertheim School of Public Health and Longevity Science, University of California San Diego, 9500 Gilman Dr., La Jolla, San Diego, CA 92093 USA; 3 The Harm Reduction Coalition of San Diego, Grossmont Center Dr, Bldg. 3, Ste. 540, La Mesa, CA 91942 USA

**Keywords:** Drug overdose, Motivational interviewing, Drug checking services, Peer-led intervention, Self-efficacy, Implementation science, Randomized controlled trial

## Abstract

**Background:**

The unpredictable and toxic nature of the unregulated drug supply poses overdose and other health risks for people who use drugs (PWUD). Drug checking services can reduce risks by identifying adulterants in individuals’ drug supplies. In the United States (U.S.), DCS are increasingly offered through community harm reduction services that provide evidence-based risk reduction services for PWUD, such as syringe services programs (SSPs). However, PWUD face several multi-level barriers to consistently utilizing DCS and engaging in post-DCS safer drug use behaviors (e.g., discard, use less, avoid using alone, etc.). Staff from a San Diego-based mobile SSP and researchers drew from Social Cognitive Theory and the Social Ecological Model to develop a motivational-interviewing adjunctive intervention (MI-CHANCE) led by peers with lived substance use experience to address multi-level barriers and promote DCS engagement and adoption of post-DCS safer drug use behaviors among PWUD.

**Methods:**

We employ a hybrid type-1 effectiveness–implementation trial with a primary goal of determining effectiveness of the MI-CHANCE adjunctive intervention in increasing DCS utilization (primary behavioral outcome) and safer drug use behaviors (secondary behavioral outcome), leading to reduced overdose risk (primary health outcome) and HIV/HCV incidence (secondary health outcome). We will recruit, consent, conduct a baseline interview, and randomize 588 PWUD who used opioids or stimulants within the week prior to receive either MI-CHANCE or an attention-control standard-of-care condition. All participants will be offered DCS following exposure to the intervention or control condition. We will conduct six-month follow-up with participants over a 30-month period to collect outcomes and hypothesized predictors, mediators and potential confounders. Our secondary goal is to assess MI-CHANCE implementation considerations guided by the RE-AIM/PRISM framework via annual in-depth interviews over the course of the trial with SSP staff (*N* = 5) and participants (*N* = 20), focusing on acceptability and feasibility, and the potential for scalability via interviews with a broad sample of diverse U.S. SSPs (*N* = 20).

**Discussion:**

This hybrid trial will advance crucial knowledge on the effectiveness and implementation of an adjunctive intervention to promote DCS engagement and safer drug use behaviors to reduce overdose risk among PWUD.

**Trial registration:**

ClinicalTrials.gov, NCT06855836. Registered February 28, 2025. https://clinicaltrials.gov/study/NCT06855836?term=NCT06855836/rank=1.

**Supplementary Information:**

The online version contains supplementary material available at 10.1186/s12889-025-24460-y.

## Background

Overdose and other health harms from unregulated drug use threaten public health in the United States (U.S.). Drug overdose is the leading cause of accidental death in the US., with over 100,000 fatalities annually between 2021 and 2023 [1]. Illicitly manufactured fentanyl (IMF) and its analogues began to infiltrate the unregulated (i.e., “street”) drug supply around 2013, accelerating the overdose crisis [2]. As a potent synthetic opioid [3], IMF causes respiratory depression and has been implicated in over 70% of drug-related deaths nationally [4]. In addition to IMF, several other emerging substances in the unregulated drug supply contribute to adverse health outcomes among people who use drugs (PWUD). For example, nitazenes are even stronger synthetic opioids that have been found in street opioids and implicated in fatal overdoses [5, 6]. Xylazine, a veterinary anesthetic and sedative increasingly detected alongside fentanyl [7], can complicate overdose [8] and cause severe skin abscesses [9]. The unregulated drug supply is constantly evolving and increasingly toxic [10], making it difficult for PWUD to anticipate the contents and effects of the drugs they consume.

Drug checking services (DCS) offer chemical analysis of drug samples and have been increasingly adopted in North America following European countries [11]. Technologies utilized in community-based DCS range from single-use test strips for targeted identification of specific substances (e.g., fentanyl test strips [FTS]) to more advanced, laboratory grade technologies (e.g., Fourier Transform Infrared spectroscopy [FTIR]) that can identify multiple substances in a single test. DCS can promote safer drug use behaviors when results indicate undesired substances in individuals’ drug samples. A recent scoping review found that 16–87% of DCS participants intended to engage in safer drug use behaviors following test results, including using less or discarding drugs completely, particularly when fentanyl was detected [12]. A Canadian study found that risk of overdose was significantly lower among participants who intended to use smaller quantities of drugs following positive FTS test results [13].

Despite high acceptability among PWUD and preliminary evidence suggesting that DCS can lead to safer drug use behaviors [14–19], multilevel barriers often prevent individuals from regularly accessing DCS and consistently adopting post-DCS safer drug use behaviors. In the U.S., community-based DCS are most commonly offered through syringe services programs (SSPs), which successfully engage PWUD in a range of evidence-based prevention services including distribution of sterile syringes and injection equipment to prevent infectious disease transmission [20] and naloxone for opioid overdose reversal [21]. Potential barriers to regular DCS utilization through SSPs include low knowledge of service availability, fear of police, stigma, fatalism or hopelessness, and competing priorities, such as the need to address withdrawal symptoms [22–25]. Barriers to adopting safer drug use behaviors following DCS use are less studied, but may include limited knowledge of and self-efficacy for safer drug use behaviors, and low expectations about the helpfulness of these behaviors.

To date, no theory-based interventions to increase DCS utilization and promote the adoption of post-DCS safer drug use behaviors have been rigorously tested. We drew from theory, literature, and community expertise to develop the “Motivational Interviewing for Community-based Harm Reduction And Drug-Checking Empowerment” (MI-CHANCE) adjunctive intervention to promote DCS utilization and post-DCS safer drug use behaviors among PWUD. Adjunctive interventions are “change methods that target recipients of a health intervention and are designed to increase motivation, self-efficacy, or capacity for initiating, adhering to, complying with, or engaging with the health intervention initially and over time.” [26] With a primary goal of determining whether the MI-CHANCE adjunctive intervention is effective, and a secondary goal of assessing implementation acceptability, feasibility and potential strategies to facilitate its adoption in other settings, we designed the hybrid type 1 trial [27] detailed in this protocol. A hybrid type-1 trial has a primary focus on determining intervention effectiveness, while exploring contexts for future intervention implementation [27, 28], an approach that accelerates the generation of evidence on both effectiveness and real-world applicability [27, 29].

This study is funded by the National Institute on Drug Abuse (grant # R33DA061260). The University of California San Diego Institutional Review Board (IRB) initially approved this study protocol on May 29, 2024 (protocol # 810285). We registered this study on ClinicalTrials.gov on February 28, 2025 (ID # NCT06855836).

## Methods/design

### Trial context and setting

Overdose deaths in California reached 11,359 in 2023, representing a 156% increase over a ten-year period [30]. San Diego is the southernmost county in California and shares one of the busiest land border crossings in the world with Tijuana, Baja California, Mexico. The White House Office of National Drug Control Policy (ONDCP) designated this border region a high intensity drug trafficking area where unregulated drugs frequently enter the U.S [31]. San Diego County (SDC) has followed the state’s increasing fatal overdose trend, with a 113% increase in deaths between 2013 and 2023.

Although prior California law had permitted DCS for identifying specific substances (e.g., fentanyl, xylazine), new legislation encouraging the expansion of DCS was passed in 2024 (AB 2136) [32]. This bill explicitly protects both DCS providers and participants from arrest for drug offenses during the drug checking process and excludes from the legal definition of drug paraphernalia equipment used to test substances for “the presence of contaminants, toxic substances, hazardous compounds, or other adulterants” [32].

The Harm Reduction Coalition of San Diego (HRCSD) is a mobile SSP established in 2018 that offers “CheckSD”, a DCS program that incorporates FTIR and immunoassay strips. HRCSD received a donation to support the acquisition of an FTIR machine in 2022 [33], officially launching the CheckSD program in 2025 with funding support from the SDC Health and Human Services Agency. For CheckSD, a trained technician prepares a small drug sample that clients bring in for testing, conducts analysis with an FTIR machine, and interprets results for clients (i.e., identifies detectable substances), complementing the process with immunoassay strips as needed to test for trace substances due to their relative sensitivity [34].

### Study participants, recruitment, and retention

Inclusion criteria for the trial are having used opioids or stimulants within the week prior to recruitment, being 18 years or older, and living in SDC with no plans to permanently move over the next 30 months. A maximum of 25% of participants will have *only* used stimulants, as overdose risk is higher among people using opioids [35]. Exclusion criteria are being enrolled in another randomized controlled trial (RCT) and having previously used FTIR-based DCS. Participants are recruited by trained and experienced bilingual research staff via street outreach in SDC where drug use is known to occur (e.g., parks, canyons, shooting galleries, motels, encampments). Research staff conduct targeted outreach using a study RV and on foot. They describe the study to potential participants, conduct a brief screener survey, invite eligible individuals to participate, and obtain informed consent. Recruitment is coordinated with HRCSD to minimize the distance consenting participants must travel to reach the CheckSD location and receive the MI-CHANCE or control condition.

We will enroll 588 PWUD participants over 18 months who will complete structured bi-annual follow-up surveys over 30 months. Statistical power calculations for the primary outcome (combined fatal and nonfatal overdose) were informed by data from an ongoing cohort study of PWUD in SDC [36]. Power was estimated using two-sided tests with α = 0.05, assuming 15% attrition over 30 months and adjusting for covariates assuming R^2^ = 0.01, yielding a final analytic sample of approximately 250 participants per arm. To minimize attrition, outreach staff record participants’ contact information, including where they typically reside and conduct quarterly check-ins to update participant contact info. Additionally, with participant consent, staff connect with participants on social media and keep record of individuals in their network who can be contacted if they cannot be located (e.g., significant others, family, friends, counselors, probation officer). A trial timeline is included in Supplementary File 1, following the “Standard Protocol Items: Recommendations for Interventional Trials” (SPIRIT) guidelines [37].

### The MI-CHANCE intervention

#### Theoretical framework

**Fig. 1:**
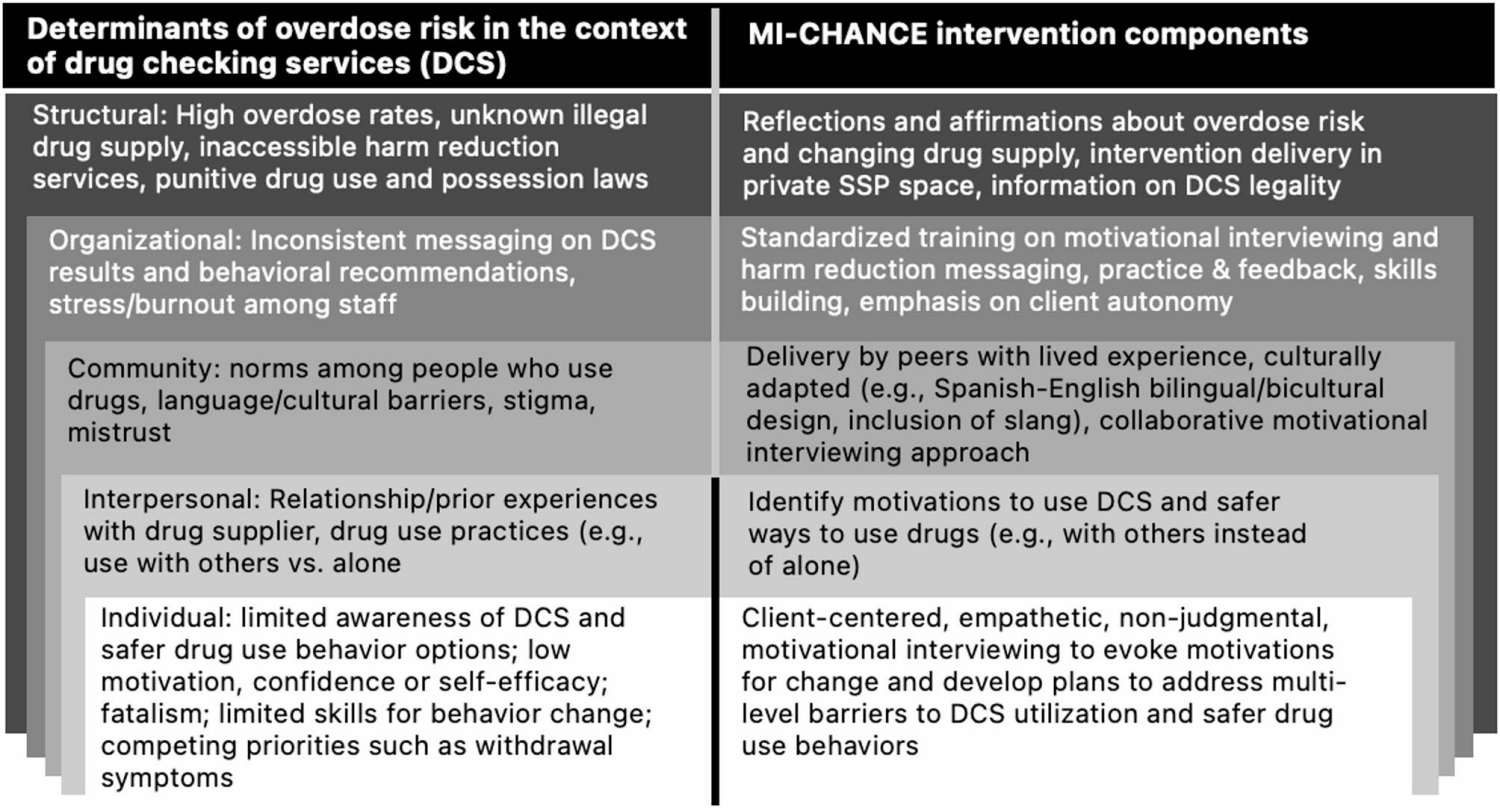
Development of the MI-CHANCE intervention: key multi-level targets and components

We developed the MI-CHANCE adjunctive intervention using the Social Ecological Model (SEM) [38, 39] as the overarching framework and a counseling approach grounded in social cognitive theory (SCT). SEM recognizes the different levels of influence affecting health outcomes (Fig. 1). The unpredictable unregulated drug supply represents a major structural determinant of overdose risk (our primary health outcome). Our intervention addresses this by encouraging use of DCS and addressing barriers at the organizational, community, and interpersonal levels. The SEM also considers the role of individual-level psychological determinants of health, which align with SCT. SCT posits that human behavior is influenced by the dynamic interaction between personal and interpersonal factors, environmental factors, and cognitive processes, such as outcome expectancies (i.e., personal beliefs about the consequences of performing a behavior), self-efficacy (i.e., confidence in one’s ability to perform a behavior) and observational learning [40]. Motivational interviewing (MI) is a behavioral counseling style that aligns with key principles of SCT. MI offers empathetic, non-judgmental guidance that supports autonomy, helps individuals explore ambivalence, and builds self-efficacy by reinforcing confidence and collaboratively identifying alternative strategies for reducing risky behaviors [41–43]. MI is well-suited for use in SSP-based interventions and has been proven effective when culturally adapted for Latinx populations in different settings [42, 44–48], who represent 35% of SDC’s population [49].

This figure outlines the development of the MI-CHANCE intervention, describing its core components and their corresponding multi-level targets informed by the Social Ecological Model.

#### Intervention adaptations

Researchers and HRCSD staff partnered to design an MI-based adjunctive intervention with the goal of encouraging uptake and continued use of DCS and adoption of post-DCS safer drug use behaviors. We structured the intervention to be facilitated in-person by peer support specialists (“peers”) who have firsthand experience with substance use, allowing them to leverage their personal knowledge and experiences to provide harm reduction services [50]. HRCSD hired peers to serve multiple roles within the organization, including DCS for the CheckSD program, and ensured that some peers spoke fluent Spanish.

The MI-CHANCE intervention is a brief, one-time, peer-led MI counseling session available in English or Spanish and offered around SDC in locations where mobile HRCSD SSP services typically operate. As part of the study, HRCSD peers receive asynchronous online and in-person training from a Motivational Interviewing Networks of Trainers (MINT) accredited trainer, as well as in-person MI-CHANCE training from members of our research team. Training includes didactic sessions and role-playing exercises. We developed a training manual for peers administering the MI-CHANCE intervention to follow four key stages using open questions, affirmations, reflections, and summaries. First, peers probe participants to share their perceptions about the street drug supply, related overdose experiences and concerns (“engage”) [51]. Second, using the “ask, offer, ask” MI approach to providing psychoeducational information [43], peers introduce the purpose, benefits, and limitations of regular DCS utilization and show participants a video FTIR drug checking demonstration (“focus”) [51]^(p3)^. Third, peers help participants explore and verbalize internal motivations for change (i.e., generate change talk, “evoke”), [51]^(p3)^ including motivations for regularly utilizing DCS and for modifying drug use behaviors according to drug checking results (e.g., avoid using the drug or use less, use more slowly, use in the presence of others, ensure naloxone availability, avoid injecting the drug, etc.). Fourth, peers support participants in integrating the regular use of DCS to their routines and planning safer drug use behaviors, by exploring the related pros and cons, identifying potential challenges and solutions and seeking verbal commitment from participants to move toward a goal, such as checking drugs before using them every time they purchase from a different source (“plan”) [51]. Importantly, participants who receive the MI-CHANCE intervention also receive standard overdose education and naloxone distribution (OEND), which includes two intranasal naloxone kits and five immunoassay strips to independently test for fentanyl and xylazine. Each MI-CHANCE session lasts approximately 30 min. Upon completion of MI CHANCE session, participants receive $10 U.S. dollars (USD) and an information card with CheckSD schedules and locations to facilitate its use during follow-up. Immediately following the MI-CHANCE session, participants are offered the opportunity to check a drug sample and to return for CheckSD services at their convenience. A Template for Intervention Description and Replication (TIDieR) checklist [52] is provided in Supplementary File 2.

#### Logic model and hypotheses

**Fig. 2:**
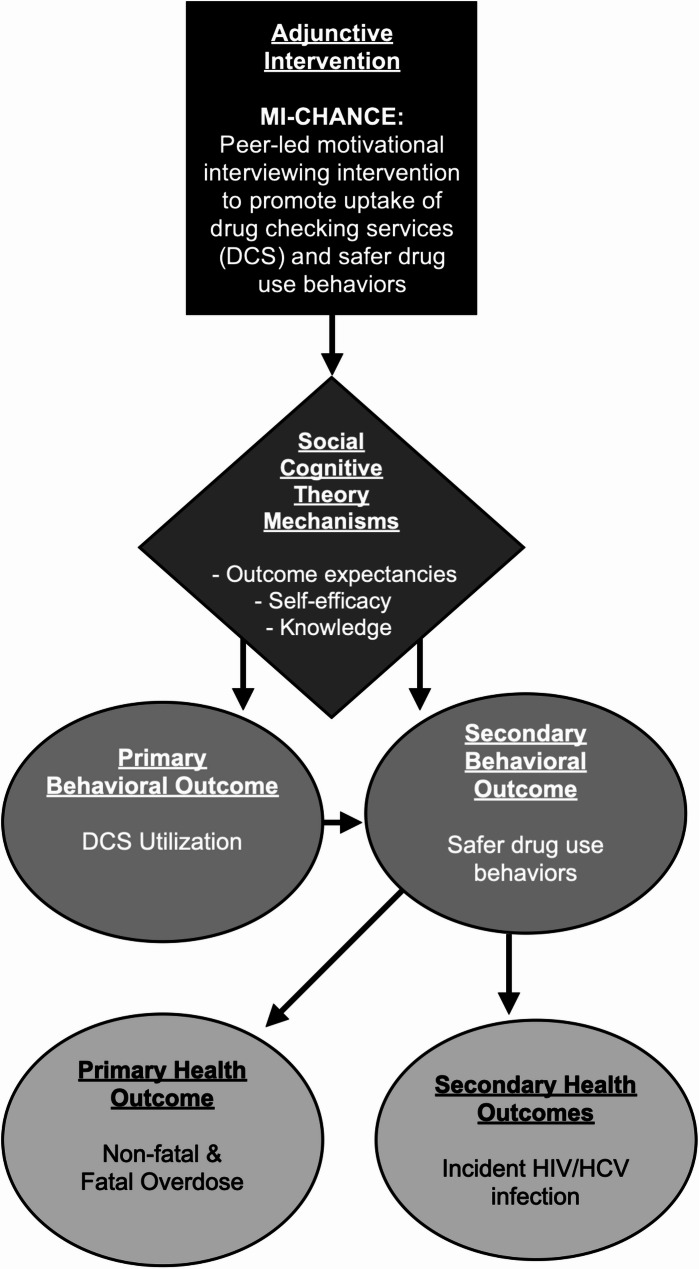
MI-CHANCE adjunctive intervention logic model

Smith et al. proposed distinctions between health interventions (those that “target recipients and have a direct, causal effect on the health outcome”) and adjunctive interventions (those that “enhance recipients behaviors to engage with the health intervention and have an indirect causal link to the health outcome via increasing the probability of recipients’ utilization and adherence to the intervention”) [26]. We hypothesize that MI-CHANCE (the adjunctive intervention) will increase DCS utilization (the health intervention) via SCT mechanisms (outcome expectancies, self-efficacy, and enhanced knowledge), ultimately leading to safer post-DCS drug use behaviors and, subsequently, lower incidence of overdose (Fig. 2). In addition, we will investigate potential reductions in human immunodeficiency virus (HIV) and hepatitis C virus (HCV) incidence as a secondary outcome, as safer drug use behaviors may include choosing alternative modes of administration to injecting (e.g., smoking) [53] to protect veins from harmful substances, thereby leading to reduced risk of HIV/HCV.

This logic model illustrates the hypothesized pathways through which the MI-CHANCE intervention influences outcomes. Peer-led motivational interviewing is hypothesized to enhance key Social Cognitive Theory constructs: participant outcome expectancies, self-efficacy, and safer drug use knowledge. These in turn lead to increased drug checking services (DCS) utilization and safer drug use behaviors. Safer drug use behaviors are hypothesized to reduce overdose risk (primary health outcome) and HIV and HCV incidence (secondary health outcome).

### Control condition

Our time-matched attention-control condition builds on the local standard of care and includes the same naloxone and immunoassay strips, plus a picture and brief description of the FTIR machine and informational videos and pamphlets on OEND and Hepatitis A and flu testing and vaccination (available in English or Spanish). Control participants are offered DCS at CheckSD following the attention-control, but do not receive any components of the active MI-CHANCE intervention condition. To match the MI-CHANCE condition, control condition sessions last approximately 30 min and participants receive $10 USD upon session completion.

### Randomization procedures

**Fig. 3:**
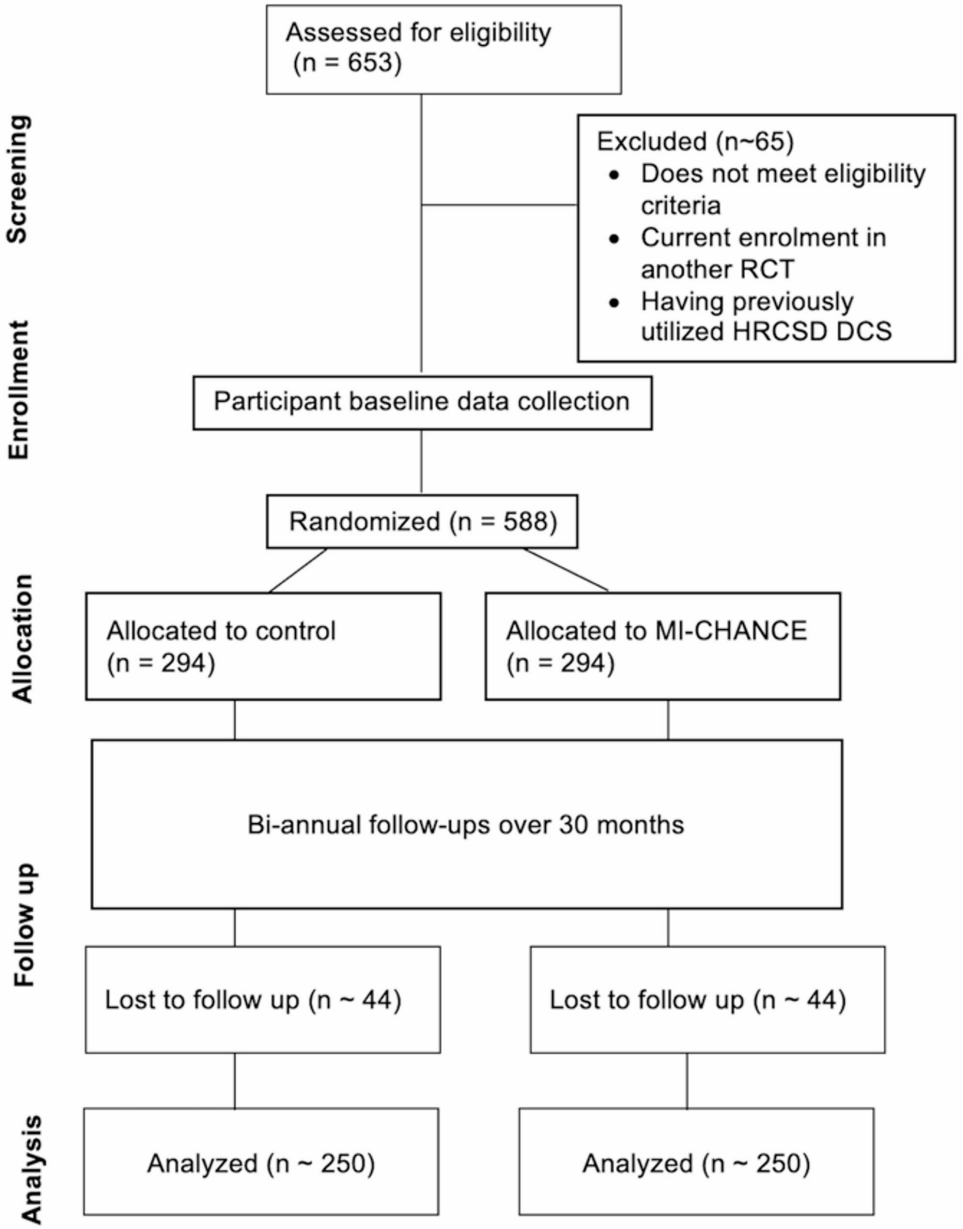
CONSORT diagram: anticipated flow of participants through the MI-CHANCE trial

To maximize reach, CheckSD is offered at six different locations across SDC, including office environments and HRCSD’s mobile outreach van. To minimize risk of contamination and bias, randomization occurs by week whereby during a given week, peers administer either the MI-CHANCE or the control condition according to predetermined randomized assignment schedules. The randomization is done in blocks of six, whereby at the end of every block of six weeks, three weeks are MI-CHANCE and three are control weeks. Randomized assignment schedules were pre-prepared using PROC PLAN in SAS^®^ 9.3 software [54]. Fig. 3 provides a consolidated standards of reporting trials (CONSORT) diagram of the participant flow through each stage of the trial [55]. A CONSORT checklist is provided in Supplementary File 3.

This flowchart depicts the expected progression of participants through the MI-CHANCE trial, including screening, enrollment, randomization, intervention allocation, follow-up, and analysis. Numbers reflect projected participant counts at each stage, with an anticipated 90% eligibility rate and a 15% attrition rate over 30 months. Abbreviations used include RCT for randomized controlled trial, HRCSD for Harm Reduction Coalition of San Diego (partnering syringe services program), and DCS for drug checking services.

### Data collection

Our primary participant data source is computer-assisted, staff-administered surveys available in English or Spanish. Surveys are conducted at baseline and approximately bi-annually using past six-month recall. Participants receive $20 USD for each completed survey. Surveys are conducted either in a private room at our study office or privately in the study RV. Survey domains include socio-demographics, past six-month sources of income, drug use (types of drugs, frequency, and routes of administration), and structural factors, including incarceration, homelessness, and the drug market (e.g., perceptions of changes in availability, purity, or price by drug type). We ask whether participants experienced an opioid overdose, how many, whether medical attention was received, and what drug(s) were used. Participants are also asked about experiences witnessing and responding to overdoses. Surveys include questions about stimulant-related toxicity and soft tissue infections. Additionally, we ask about knowledge, access to, and utilization of harm reduction and healthcare services (e.g., SSPs, naloxone, medications for opioid use disorder [MOUD], vaccinations) and obtain consent for release of medical records and data linkage. Finally, participants are offered HIV and HCV testing at baseline and follow-up.

### Effectiveness outcomes and additional measures

#### Proximal behavioral outcomes

Our primary behavioral outcome is past six-month frequency of CheckSD utilization at each follow-up visit. The outcome measure is discrete (i.e., the number of times participants utilized CheckSD in the past 6 months).

Our secondary behavioral health outcomes are post-DCS safer drug use behaviors. These will be binary measures and include any of the following: avoiding fentanyl, using lower amounts, using more slowly, doing “tester shots” (i.e., injecting a small amount of the drug to gauge effects before injecting the rest), checking drugs before instead of after using drugs, not injecting, not using drugs while alone, carrying naloxone and/or using drugs with others who carry naloxone, and engaging in drug treatment (e.g., MOUD).

#### Distal health outcomes

Our primary effectiveness outcome variable is combined incidence of fatal and nonfatal overdose, determined by non-nominal record-linked data and self-report. The SDC medical examiner will confirm fatal overdoses based on ICD-10 underlying cause-of-death codes X40-44 (unintentional) and Y0-Y14 (undetermined intent). Nonfatal overdose will be ascertained via self-report at semi-annual follow-up interviews with participants and supplemented by Emergency Medical Services (EMS) data. The survey measure asks participants how many times in the past six months they experienced an opioid overdose, meaning a “situation where you passed out and could not wake up or your lips turned blue.”

Our secondary effectiveness outcome variable is risk rates of combined HIV/HCV incidence, based on serological tests (Medmira^®^ Miriad combined HIV/HCV immunoassay rapid tests) [56]. Positive results will be confirmed with a second test using Orasure^®^ [57]. Pre- and post-test counseling will be provided following national guidelines. Participants testing positive are referred to local community clinics for follow-up care.

### Mediators

#### Social cognitive theory mechanisms

We posit that the relationship between exposure to the MI-CHANCE intervention and our behavioral health outcomes (CheckSD use and safer drug use behaviors) will be mediated by SCT mechanisms, including outcome expectancies, self-efficacy, and knowledge. Outcome expectancies are measured with a series of binary yes/no questions asking participants about anticipated outcomes related to DCS (e.g., *I will know what to do if my drug checking results indicate my drugs are contaminated*, or, *Drug checking with CheckSD will help me to avoid overdose*). A composite score will be calculated based on the number of affirmative responses, with higher scores indicating more positive outcome expectancies. Self-efficacy measures were adapted from the general self-efficacy scale (GSE-6) [58] and the safe injection self-efficacy scale [59, 60]. These questions ask participants to indicate on a Likert scale from 1) *absolutely sure I cannot*, to 4) *absolutely sure I can*, the certainty with which they can engage in drug checking while facing different scenarios, such as when they get drugs from a new supplier or when they are experiencing withdrawal. A self-efficacy score is calculated as the average of all item responses, with higher scores indicating greater self-efficacy. DCS knowledge is measured with a series of binary yes/no questions, including whether they participants know where to go to utilize DCS, differences between immunoassay and FTIR DCS, the type of results they can get from CheckSD, etc. DCS knowledge will also be calculated as a composite score.

### Data analysis

We will use an intent-to-treat approach. For each outcome and semi-annual time point, cross-tabulations will be generated using proportions for categorical variables and means, medians and IQRs for continuous variables. Hypothesis tests will use mainly generalized linear mixed models with random slopes and intercepts for each subject to account for unmeasured baseline differences, variable visit times and correlations from repeated measures. We assume an autoregressive structure (i.e. subject-specific variances and correlations decline exponentially with time). Analyses will consider covariates such as CheckSD location, prior use of SSPs or immunoassay DCS (yes vs. no), age, sex, race/ethnicity, education, drug of choice, housing status, recent incarceration and income. The specific covariates to control for will be selected following the methodology outlined by VanderWeele’s “Principles of confounder selection” [61].

To test our hypothesis that MI-CHANCE will lead to greater frequency of DCS utilization, will use mixed effects negative binomial regression, with *number of times CheckSD was used in the past six months* as the outcome and *time* (Baseline, 6-months, 12-months…30-months), *intervention group*, and time*group as main fixed effects. We expect the interaction to be significant but not confounding (i.e., differing in magnitude but not direction), in which case the main effect of the intervention on the outcome will be interpreted directly from the full model. If the interaction is significant and confounding, the intervention effect on the outcome will be evaluated in separate models at each time point.

To test our hypothesis that MI-CHANCE will lead to greater likelihood of post-DCS safer drug use behaviors, we will use mixed effects log binomial regressions or Poisson regressions, with each safer drug use behavior as the outcome and *time*, *intervention group*, and time*group as the fixed main effects. We expect the interaction to be significant but not confounding. To determine whether the relationship between intervention group and post-DCS safer drug use behaviors is mediated by (a) outcome expectancies, (b) self-efficacy related to safer drug use, and (c) knowledge, we will utilize equations provided by Geldof et al. [62] to evaluate and interpret our mediation model.

To test our hypothesis that combined fatal and non-fatal overdose rates will be significantly lower in the MI-CHANCE vs. control group, we will conduct either a mixed effects Poisson regression or a negative binomial regression (if over-dispersion is present), with count of overdoses over 30 months’ follow-up as the outcome, natural log of time at risk as an offset term and intervention group as the primary fixed predictor. Random intercepts will be used to account for any unmeasured baseline differences between subjects. The outcome will be assessed cumulatively over 30 months of follow-up; however, we will also conduct a separate mixed effects repeated measures analysis, with number of overdoses in the past 6 months as the outcome and *time*, *intervention group* and the interaction between the two as main fixed effects to allow us to estimate how the overdose rate changed over time and whether the change was directionally the same for the two groups or different.

To test our secondary hypothesis that HIV/HCV incidence will be significantly lower among MI-CHANCE vs. control group participants, we will conduct a Cox regression with the time to HIV/HCV seroconversion as the outcome, assuming the cases occurred at the midpoint between last negative and first positive HIV/HCV test. Non-cases will be right censored. *Intervention group* will be the primary fixed predictor and both fixed and time-varying potential confounding variables will be considered as covariates.

## Implementation assesment and methods

### Overview

Our assessment of MI-CHANCE implementation addresses inward- and outward-looking research questions that will be examined using multiple methods. First, we will conduct in-depth interviews with HRCSD staff and MI-CHANCE trial participants to gather perspectives on intervention acceptability and feasibility (primary outcomes) and sustainability (secondary outcome). Second, we will quantitatively and qualitatively assess intervention fidelity with HRCSD staff and collect implementation costs to inform potential intervention scale-up. Third, we will explore perceptions of scalability and potential adoption via interviews with various SSPs across the U.S.

### Theoretical framework

**Fig. 4:**
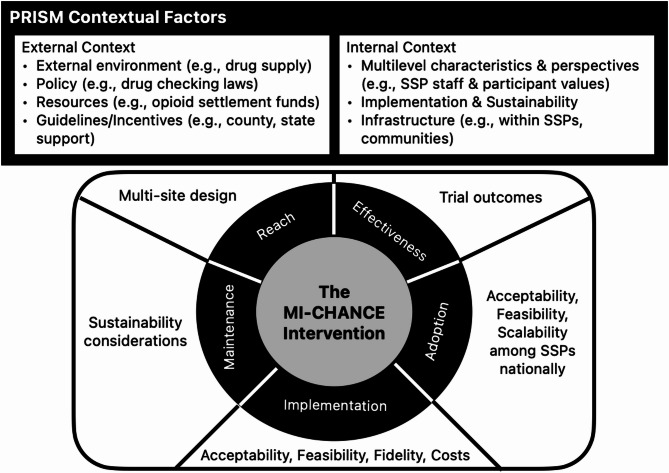
RE-AIM/PRISM framework guiding the Mi-CHANCE hybrid type 1 effectiveness-implementation trial

Our implementation assessment is guided by the Reach, Effectiveness, Adoption, Implementation, and Maintenance Framework and the Practical, Robust Implementation and Sustainability Model (RE-AIM/PRISM, Fig. 4) [29, 63]. This integrated framework is particularly well-suited to our study because it emphasizes both intervention effectiveness—which we will rigorously assess in our trial—and the real-world implementation determinants that shape acceptability, feasibility, sustainability, and eventual scale-up. RE-AIM provides a structured approach to examining various dimensions of implementation across individual and organizational levels. PRISM complements this by highlighting contextual influences, such as organizational characteristics, external policies, and environmental factors, that shape how implementation unfolds in real-world settings. These contextual elements are especially important for SSPs, which operate in dynamic environments shaped by stigma, limited resources, and variable policy landscapes.

This figure illustrates the application of the RE-AIM (Reach, Effectiveness, Adoption, Implementation, Maintenance) and PRISM (Practical, Robust Implementation and Sustainability Model) frameworks to guide the MI-CHANCE trial. The framework highlights key external and internal contextual factors that may influence both effectiveness and implementation outcomes of the peer-delivered motivational interviewing intervention in syringe services programs (SSPs).

### Inward-looking assessment

#### Participants and recruitment

For the inward-looking implementation assessment, we will recruit 3–5 HRCSD staff for yearly in-depth interviews over the four-year trial period to explore implementation considerations. We will also annually recruit a subset of 20 participants who received the MI-CHANCE intervention for qualitative interviews. Of 20 yearly participants for qualitative interviews, ten will be newly recruited and ten will be re-engaged over the four-year trial to include a longitudinal perspective of MI-CHANCE sustainability, yielding a total of 80 interviews among 50 participants [64]. Participants will be purposively recruited to achieve a variety of perspectives based on overdose experiences (none, one, two or more), patterns of DCS use (never/once/frequent), gender, race/ethnicity, language preference, drug use, justice involvement, and housing status [65].

#### Data collection, measures and analysis

##### Acceptability, feasibility, and sustainability

We will develop in-depth, semi-structured interview guides with open-ended questions and detailed probes based on the RE-AIM/PRISM framework. Following informed consent, participants (HRCSD staff and MI-CHANCE participants) will undergo hour-long interviews in English or Spanish. Interviews will focus on primary implementation outcomes of acceptability and feasibility [66]. Acceptability refers to the perception that MI-CHANCE is “agreeable, palatable, or satisfactory,” while feasibility is the extent to which MI-CHANCE can be successfully utilized or implemented within a given setting [67]. Interview questions will prompt participants to reflect on MI-CHANCE acceptability and feasibility by exploring factors such as the time commitment, the relevance and usefulness of the content, the appropriateness of the format and delivery method, ease of delivery or participation, cultural or linguistic fit, etc. With HRCSD staff we will also discuss any personnel, infrastructure, or funding constraints to implementation and ask them to describe how MI-CHANCE fits or interferes with their daily SSP activities and their perceptions of whether the intervention might be institutionalized (i.e., sustainability––secondary outcome). With MI-CHANCE participants, additional questions will explore barriers and facilitators of DCS utilization and the extent to which the MI-CHANCE intervention addresses these, as well as perceptions of the likelihood of sustained engagement with CheckSD. HRCSD will receive $200 USD compensation annually for staff participation in interviews and MI-CHANCE participants will receive $20 USD per interview.

##### Fidelity

We will track fidelity to the MI-CHANCE intervention at the peer level. Fidelity will be monitored by recording and reviewing a sample of MI sessions using an MI fidelity checklist adapted to MI-CHANCE stages, and by reviewing process forms, which will be completed by peers for each participant encounter (see Supplementary Files 4 and 5 for fidelity checklist and process forms). Research staff will review process forms for consistency regarding content, coverage, and duration and will rate MI sessions based on content, quality, and participant responsiveness using the fidelity checklist.

##### Costs

Finally, we will also collect information about costs associated with MI-CHANCE implementation to inform adoption and future cost-effectiveness analyses. Via a micro-costing analysis, we will determine total, component-specific, and per-participant costs of both FTIR-based DCS and MI-CHANCE to estimate the relative and additional cost of MI-CHANCE versus DCS alone. Micro-costing is a uniformed and standardized method of identifying detailed costs so these can be used to compare estimates across studies [68, 69]. We will cost the intervention from the perspective of the SSP and intervention components will be split into fixed and variable costs. We will develop process forms to be completed by HRCSD staff to track time and resource utilization to facilitate cost calculations.

##### Analysis

All interviews will be recorded, transcribed, and translated as necessary. For each set of interviews, researchers will develop a preliminary codebook reflecting considerations for MI-CHANCE implementation drawing from RE-AIM/PRISM constructs and key implementation outcomes as well as SEM levels of influence. We will employ a hybrid inductive/deductive thematic analysis approach whereby themes are categorized according to our theoretical frameworks, while allowing for additional codes based on emerging topics [70, 71]. In addition to overarching themes, we will also compare sub-groups to determine whether there are distinct findings (e.g., differences in experience based on types of drugs used for MI-CHANCE participants).

### Outward-facing assessment

#### Participants and recruitment

For the outward-looking implementation assessment, we will recruit 1–3 staff members from ~ 20 SSPs across the U.S. purposively sampled for DCS experience (i.e., nascent vs. established programs), geographic diversity (e.g., West, Midwest, South, Northeast), and organization size and type (e.g., health department vs. NGO). We will recruit SSPs using our professional connections, recommendations from enrolled SSPs (i.e., snowball sampling) [72], and the North American Syringe Exchange Network’s publicly available online directory [73]. We will contact SSP directors to briefly explain the study procedures; interested individuals will be eligible if they are ≥ 18 years of age, report familiarity with their SSP’s DCS, and provide informed consent.

#### Data collection and analysis

Staff representing U.S. SSPs will be asked to participate in a brief survey to collect basic organizational characteristics, funding sources, days/hours of operation, key services offered, and additional DCS contextual factors. Following the survey, we will conduct virtual interviews focused on the scalability of MI-CHANCE. Scalability refers to the potential for an intervention to be expanded successfully. Scale-up is defined as “the deliberate effort to increase the impact of successfully tested health interventions so as to benefit more people and to foster program development on a lasting basis“ [74]. Using open-ended questions, we will explore the local drug supply context, overdose risks and responses, existing DCS services and related challenges and opportunities, organizational and external factors that may influence the feasibility, acceptability, and sustainability of MI-CHANCE, and other factors informed by our trial results. We will also assess the perceived fit of the intervention within existing services and explore SSP staff perspectives on potential implementation strategies that could support MI-CHANCE adoption (e.g., adapt and tailor the intervention, audit and feedback, incorporate external implementation advisors or prepare internal implementation champions, etc.) [75]. Interviews will last approximately one hour and SSPs with staff participation will receive $200 USD. We will analyze interviews with the same process described for the inner-looking assessment, exploring potential thematic differences based on SSP characteristics (e.g., prior DCS experience, geographic region, and SSP type).

## Discussion

The MI-CHANCE hybrid trial appears to be the first to test the efficacy and identify implementation determinants of a theory-based adjunctive intervention to potentiate community-based DCS for the prevention of overdose and other harms related to the use of unregulated drugs. This study stands at the intersection of clinical efficacy and implementation science, offering crucial insights not only into whether the MI-CHANCE adjunctive intervention is effective but also into how it could be successfully adopted and sustained in various real-world community settings. Hybrid studies allow for the accelerated transition of an evidence-based intervention into communities where solutions are urgently needed, compared with traditional approaches that assess efficacy and implementation sequentially [27, 28]. The importance of this study lies in the continued public health crisis related to the volatile unregulated drug supply. Community-based harm reduction strategies such as DCS are essential tools in mitigating drug use risks and have contributed to recent decreases in fatal overdose rates [76]. However, their reach and impact remain limited due to various multi-level barriers to uptake and consistent utilization for PWUD. This trial aims to address these challenges by testing an innovative, theoretically grounded adjunctive intervention that targets environmental, social, and psychological factors influencing DCS engagement and drug use behaviors among PWUD.

From an implementation science perspective, the MI-CHANCE trial offers a unique opportunity to explore the role and implementation of adjunctive interventions in the novel and understudied setting of community-based harm reduction organizations. Understanding the determinants of MI-CHANCE acceptability, feasibility, and scalability in these settings will be crucial for guiding future efforts to optimize DCS effectiveness with adjunctive motivational interviewing and findings will likely carry important implications for the integration of other evidence-based approaches into harm reduction settings. By studying MI-CHANCE implementation based on the practical experiences of local SSP staff and diverse participants and exploring perspectives across a variety of U.S. SSPs, the study will provide invaluable insights into how such interventions could be adapted to fit different contexts. This will help ensure that DCS programs are culturally relevant and responsive to the specific needs of local organizations and the communities they serve.

### Potential limitations

There are several limitations inherent in the study design presented in this protocol. First, there are two possible sources of contamination: peers with MI-CHANCE training and crosstalk between subjects assigned to different conditions. To mitigate contamination from peers, we will provide rigorous training and implement fidelity checks throughout the study. To assess the extent of crosstalk between participants, we will ask participants in follow-up surveys if they have discussed the MI-CHANCE intervention with other people and, in follow-up interviews with a subset of participants, inquire about whether they have discussed the intervention with other PWUD and explore the content of those discussions.

Second, generalizability of our results is limited given our focus on a single SSP in SDC. Our outward-facing implementation assessment addresses this by exploring the possibility of scaling MI-CHANCE to SSPs with differing characteristics and contexts and potential strategies for doing so. One important limitation, however, is that DCS are not explicitly legal in every US state [77], and even in states where DCS exist in legal grey areas, limited clarity in legal status is often cited as a significant structural-level barrier to implementation [12, 78]. If MI-CHANCE proves effective, opportunities for scale-up will be constrained to states where DCS implementation is legally or practically feasible.

Finally, as a hybrid type-1 trial, our primary focus is effectiveness of the MI-CHANCE adjunctive intervention. If effective, additional research will be required to determine cost-effectiveness and develop and test strategies to adapt MI-CHANCE to other contexts and facilitate its implementation.

The MI-CHANCE hybrid type-1 effectiveness-implementation trial holds significant promise for advancing both the scientific understanding of adjunctive interventions and the practical implementation of these services in community-based harm reduction settings. By testing the intervention’s effectiveness and identifying the critical factors for its successful implementation, this study will pave the way for more effective, equitable, and scalable approaches to addressing overdose and other urgent public health issues associated with unregulated drug use amidst a toxic drug supply.

## Supplementary Information

Below is the link to the electronic supplementary material.

Supplementary Material 1

Supplementary Material 2

## Data Availability

No datasets were generated or analysed during the current study.

## Abbreviations

U.S.: United States of America
IMF: Illicitly manufactured fentanyl
PWUD: People who use drugs
DCS: Drug checking services
FTS: Fentanyl test strips
FTIR: Fourier transform infrared spectroscopy
SSP: Syringe services program
MI-CHANCE: Motivational interviewing for community-based harm reduction and drug-checking empowerment
IRB: Institutional review board
ONDCP: White House Office of National Drug Control Policy
SDC: San Diego County
HRCSD: Harm Reduction Coalition of San Diego
RCT: Randomized controlled trial
SPIRIT: Standard protocol items: recommendations for interventional trials
SEM: Social ecological model
SCT: Social cognitive theory
MI: Motivational interviewing
MINT: Motivational interviewing networks of trainers
OEND: Overdose education and naloxone distribution
USD: U.S. dollars
TIDieR: Template for intervention description and replication
HIV: Human immunodeficiency virus
HCV: Hepatitis C virus
CONSORT: Consolidated standards of reporting trials
MOUD: Medications for opioid use disorder
EMS: Emergency medical services
GSE-6: General self-efficacy scale
RE-AIM/PRISM: Reach, effectiveness, adoption, implementation, and maintenance framework and practical, robust implementation and sustainability model
