# Supplementary material for: Motivational interviewing to increase drug checking and reduce overdose rates among people who use drugs: protocol for a hybrid type 1 effectiveness–implementation trial of an adjunctive intervention
Source: BMC Public Health. 2025 Sep 30;25:3228. doi: 10.1186/s12889-025-24460-y (PMC12487341; doi:10.1186/s12889-025-24460-y)
Supplement: Supplementary file 1 — Supplementary Material 1 [file 12889_2025_24460_MOESM1_ESM.doc]

Supplementary File 1. Standard Protocol Items: Recommendations for Interventional Trials (SPIRIT) Diagram for the MI-CHANCE Trial

|  | **Enrolment** | **Allocation** | **Post-allocation** | | | | | **Post-trial** |
| --- | --- | --- | --- | --- | --- | --- | --- | --- |
| **TIMEPOINT**** | ***Pre-Allocation*** | **0** | ***6 months*** | ***12 months*** | ***18 months*** | ***24 months*** | ***30 months*** | ***36***  ***months*** |
| **ENROLMENT:** |  | | | | | | |  |
| **Eligibility screen** | X |  |  |  |  |  |  |  |
| **Informed consent** | X |  |  |  |  |  |  |  |
| **Allocation** |  | X |  |  |  |  |  |  |
| **INTERVENTIONS:** |  | | | | | | |  |
| **MI-CHANCE intervention** |  | X |  |  |  |  |  |  |
| **Standard-of-Care Attention-control** |  | X |  |  |  |  |  |  |
| **ASSESSMENTS:** |  | | | | | | |  |
| **Baseline** | X |  |  |  |  |  |  |  |
| **Outcome variables:**  **(Primary: Overdose, Secondary: Safer drug use behaviors, Frequency of using CheckSD)** |  |  | X | X | X | X | X |  |
| **Mediating variables: (knowledge, outcome expectancies, self-efficacy)** |  |  | X | X | X | X | X |  |
| **Implementation inner-setting outcomes** |  | X |  | X |  | X |  |  |
| **MI-CHANCE and CheckSD costs** |  |  | X |  |  |  | X |  |
| **Implementation outer-setting determinants** |  |  |  |  |  | X | X | X |
